# Machine Learning Approaches for Predicting Bisphosphonate-Related Osteonecrosis in Women with Osteoporosis Using *VEGFA* Gene Polymorphisms

**DOI:** 10.3390/jpm11060541

**Published:** 2021-06-10

**Authors:** Jin-Woo Kim, Jeong Yee, Sang-Hyeon Oh, Sun-Hyun Kim, Sun-Jong Kim, Jee-Eun Chung, Hye-Sun Gwak

**Affiliations:** 1 Department of Oral and Maxillofacial Surgery, School of Medicine, Ewha Womans University, Seoul 07985, Korea; jinu600@gmail.com (J.-W.K.); lovegusdl@gmail.com (S.-H.K.); sjsj7777@ewha.ac.kr (S.-J.K.); 2 Graduate School of Pharmaceutical Sciences, College of Pharmacy, Ewha Womans University, 52 Ewhayeodae-gil, Seodaemun-gu, Seoul 03760, Korea; jjjhello1@naver.com; 3 Institute of Pharmaceutical Science and Technology, College of Pharmacy, Hanyang University, 55 Hanyangdaehak-ro, Sangnok-gu, Ansan 15588, Korea; dprtmvkdlvm@naver.com

**Keywords:** bisphosphonate-related osteonecrosis, VEGFA, gene polymorphism, machine learning

## Abstract

Objective: This nested case–control study aimed to investigate the effects of *VEGFA* polymorphisms on the development of bisphosphonate-related osteonecrosis of the jaw (BRONJ) in women with osteoporosis. Methods: Eleven single nucleotide polymorphisms (SNPs) of the *VEGFA* were assessed in a total of 125 patients. Logistic regression was performed for multivariable analysis. Machine learning algorithms, namely, fivefold cross-validated multivariate logistic regression, elastic net, random forest, and support vector machine, were developed to predict risk factors for BRONJ occurrence. Area under the receiver-operating curve (AUROC) analysis was conducted to assess clinical performance. Results: The *VEGFA* rs881858 was significantly associated with BRONJ development. The odds of BRONJ development were 6.45 times (95% CI, 1.69–24.65) higher among carriers of the wild-type rs881858 allele compared with variant homozygote carriers after adjusting for covariates. Additionally, variant homozygote (GG) carriers of rs10434 had higher odds than those with wild-type allele (OR, 3.16). Age ≥ 65 years (OR, 16.05) and bisphosphonate exposure ≥ 36 months (OR, 3.67) were also significant risk factors for BRONJ occurrence. AUROC values were higher than 0.78 for all machine learning methods employed in this study. Conclusion: Our study showed that the BRONJ occurrence was associated with *VEGFA* polymorphisms in osteoporotic women.

## 1. Introduction

Bisphosphonates are widely used to treat various bone diseases, including osteoporosis and cancer-induced bone metastasis. Osteonecrosis of the jaw (ONJ) is a rare but severe adverse effect of bisphosphonate treatment [1]. The clinical manifestations of ONJ include the presence of exposed bone in the maxillofacial area for more than 8 weeks in patients with current or previous bisphosphonate administration, in the absence of head and neck radiation therapy [2]. Since BRONJ was first described in 2003, denosumab, which is a new antiresorptive; tyrosine kinase inhibitors; mammalian target of rapamycin inhibitors; monoclonal antibodies; radiopharmaceuticals; selective estrogen receptor modulators; and immunosuppressants have been implicated in ONJ [3]. Despite an enormous amount of research that has been reported, its pathogenesis is poorly understood; current theories include suppression of bone remodeling, inflammation, altered gingival fibroblast function, impaired immune function, and inhibition of angiogenesis [4,5].

The majority of ONJ cases occur after dental surgery, such as tooth extraction [6], and thus wound healing processes may be involved. Complementary treatment, such as laser, ozone therapy and application of platelet concentrates in solid and liquid form, would allow both to prevent ONJ and improve healing after surgical treatment of bone lesions [7,8,9]. Blood vessel growth is essential for initiating and sustaining wound healing. Inhibition of healing in hard and soft tissues, as well as the consequent effects on the vasculature, are presumed to have anti-angiogenic effects [10]. Thus, it is assumed that ONJ may develop, at least in part, due to the effect of bisphosphonates on angiogenic gene expression in healing tissues.

Vascular endothelial growth factor A (VEGF-A) is one of the most potent pro-angiogenic factors involved in wound healing [11]. During angiogenesis, endothelial cell proliferation is required to form new vessels, and VEGF-A promotes proliferation and migration of vascular endothelial cells. VEGF-A reduction is often observed in patients with ONJ [12]. Bisphosphonates are known to suppress angiogenesis following tooth extraction [13]. Additionally, microvascular defects associated with BRONJ lesions have been reported [14]. However, the pathophysiology associated with BRONJ development is still unclear, resulting in uncertainty regarding the genetic factors associated with BRONJ.

Several studies have reported BRONJ-associated genes. Through genome-centered studies, including genome-wide association studies or exome sequencing studies, associations of *CYP2C8, PPARG, RBMS4, ASRD, SLC25A5, CCNYL2,* and *SIRT1* with BRONJ have been reported [15,16,17]. Additionally, *FDPS, HLA-DRB1, HLA-DQB1, CYP19A1,* and *VEGF* have shown significant associations with BRONJ through gene-centered studies such as single-nucleotide polymorphism (SNP) analyses [18]. However, many of these relevant studies enrolled healthy controls without taking bisphosphonate [17,19]. In other studies, all of the participants comprised bisphosphonate users, but they received treatment only with zoledronic acid among various bisphosphonates for both solid tumors and multiple myeloma [17,20,21,22]. Moreover, most such studies investigated oncology patients, and to our knowledge, there are no publications reporting related studies of patients with osteoporosis.

Machine learning is a field of study that gives computers the capability to learn without being explicitly programmed. Machine learning has been widely used for prediction in several areas, including medical fields [23]. With huge progress in machine learning techniques, there have been several studies using machine learning in dental and maxillofacial fields, such as periodontology, endodontics, orthodontics, radiology, and dental and maxillofacial surgery [24,25,26,27]. However, the use of machine learning methods to predict BRONJ has never been reported.

Therefore, we aimed to evaluate the association between *VEGFA* polymorphisms and bisphosphonate-related ONJ occurrence in osteoporosis patients taking bisphosphonates, and we used supervised machine learning to build predictive models for BRONJ occurrence.

## 2. Materials and Methods

### 2.1. Patients and Data Collection

This prospective, nested case-control study was conducted from January 2014 through December 2018 at Ewha Womans University Mokdong Hospital. Patients with current or previous bisphosphonate use who were scheduled for dentoalveolar surgery were enrolled in the study. Eligible patients were those older than 50 years diagnosed with osteoporosis by a medical doctor. Patients with any history of head and neck radiation or tumors necessitating antiresorptive drug administration were excluded. BRONJ was diagnosed by oral surgeons according to the guidelines of the American Association of Oral and Maxillofacial Surgeons [28]. The study was approved by the institutional review board of Ewha Womans University Mokdong Hospital (IRB number: 14-13-01) and conducted in accordance with the Declaration of Helsinki. Informed consent was obtained from all patients before their participation in the study. Clinical information was recorded and collected from electronic medical records. The collected information included patients’ age, gender, comorbidities, and duration of bisphosphonate use.

### 2.2. Genotyping

Saliva samples were collected for genotyping using the tube format (OG300) of the Oragene^®^·DNA Self-Collection Kit (DNAgenotek, Kanata, ON, Canada). The samples were incubated at 50 °C for 2 h before DNA extraction, and genomic DNA extraction was performed according to the manufacturer’s instructions. The genetic information of the *VEGFA* SNPs was obtained from Haploreg 4.1, the SNP database of the National Center for Biotechnology Information. Eleven *VEGFA* SNPs (rs2010963, rs699947, rs10434, rs25648, rs3024987, rs3025022, rs3025035, rs3025039, rs998584, rs6905288, and rs881858) were selected and genotyped to investigate their associations with BRONJ development [29,30,31,32,33,34,35,36]. These SNPs were analyzed by SNaPShot Multiplex kits (ABI, Foster City, CA, USA) according to the manufacturer’s instructions. Genotyping was performed by a single-base primer extension assay using SNaPShot multiplex kits (ABI) or TaqMan genotyping assays using a real-time polymerase chain reaction system (ABI 7300, ABI) according to the manufacturer’s instructions.

### 2.3. Statistical Analysis and Machine Learning Methods

The chi-squared test was used to compare categorical variables, and Student’s *t*-test was used to compare continuous variables between the case and control groups. Multivariable logistic regression analysis was used to examine independent risk factors for BRONJ. Factors that had *p* values < 0.05 in the univariate analysis were included in multivariate analysis. Odds ratios (ORs) and adjusted odds ratios (aORs) were calculated from univariate and multivariate analyses, respectively. Attributable risk (%) was calculated as follows: (1-1/aOR) × 100.

**Figure 1:**
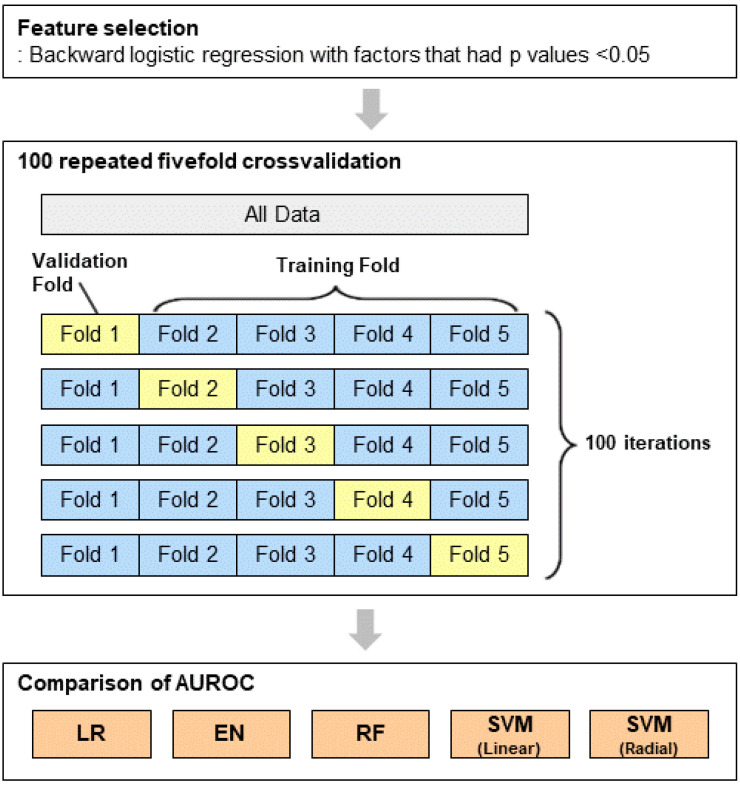
Flow chart of the machine learning approaches.

Machine learning algorithms were developed to predict risk factors for BRONJ occurrence (Figure 1). Fivefold cross-validated multivariable logistic regression, elastic net, random forest (RF), and support vector machine (SVM) classification models were utilized. All the methods were implemented using the R package caret. For cross-validation, the dataset was randomly divided into five equal subsets. After partitioning one data sample into five subsets, we selected one subset for model validation, while the remaining subsets were used to establish machine learning models. Each cross-validation iteration was repeated 100 times to evaluate the power of the machine learning models. In elastic net, the gird-search value for λ and α, which controls the weight that is given to the penalty and the weight given to ridge or lasso penalty, respectively, was varied. In terms of RF, the mtry, the number of randomly selected predictors, was tested. For SVM, we used the linear and radial kernel functions, and the cost and sigma were optimized.

To assess the ability of the constructed models for BRONJ occurrence, we analyzed the area under the receiver-operating curve (AUROC) and its 95% confidence interval (CI) of each model. All statistical tests were conducted with a two-tailed alpha of 0.05. The data were analyzed using Statistical Package for Social Sciences Version 20.0 for Windows (SPSS, Chicago, IL, USA). Machine learning algorithms were constructed using R software version 3.6.0 (R Foundation for Statistical Computing, Vienna, Austria).

## 3. Results

Of the 149 patients screened for inclusion in this study, 24 were excluded for the following reasons: 20 patients with additional indications other than osteoporosis, 2 patients without clinical information, and 2 men. A total of 125 patients were included in the final analysis. As shown in Table 1, 58 patients (46.4%) developed BRONJ after dental procedures. The mean age of study patients was 72.9 ± 9.4 years, and 19 patients were under 65 years of age. Hypertension was more common among cases than controls (62.4% versus 41.8%, *p* < 0.05). The proportion of patients treated for 36 months or longer was higher in the case group than the control group (*p* < 0.01) (Table 1).

Among the 11 *VEGFA* SNPs evaluated, all of the allele frequencies were consistent with the Hardy–Weinberg equilibrium. Univariate analysis revealed rs10434 (A > G) and rs881858 (G > A) as significantly associated with BRONJ development. Variant homozygous carriers (GG) of rs10434 developed BRONJ more often than those with other genotypes. Wild G allele carriers of rs881858 had a higher risk of BRONJ development than those with other genotypes (Table 2).

After adjusting for demographic variables with *p* < 0.05, we found that the odds of BRONJ development were about 6.45 times higher among the G allele carriers of rs881858 than the odds among variant homozygote carriers (*p* < 0.01). The rs10434 polymorphism did reach the marginal significance after adjusting for covariates. Additionally, in terms of BRONJ development, patients who were treated for longer than 36 months and those who were older than 65 years of age had ORs of 3.67 and 16.05, respectively (Table 3). The attributable risk of the rs881858 polymorphism was 84.5%. The AUROC of logistic regression was 0.818 (95% CI, 0.736–0.901).

After we performed fivefold cross-validated multivariate logistic regression, elastic net, RF, and SVM models (linear kernel and radial kernel), the AUROC values (mean, 95% CI) across 100 random iterations showed the clinical performance as follows: fivefold cross-validated multivariable logistic regression (0.788, 0.702–0.875), elastic net (0.788, 0.702–0.875), RF (0.781, 0.694–0.878), linear kernel SVM (0.786, 0.694–0.878), and radial kernel SVM (0.793, 0.706–0.879).

## 4. Discussion

The main finding of this study was that, in terms of BRONJ development, the odds of wild-G allele carriage of rs881858, with an OR of 6.45 and an attributable risk of 84.5%, were higher than the odds of variant homozygosity. After adjusting for covariates, we found that patients with the GG rs10434 genotype showed an approximately 3.16-fold (95% CI, 0.97–10.31) increased risk of BRONJ compared with those with the A allele. Among demographic variables, age ≥ 65 years and treatment duration ≥ 36 months were significant risk factors for BRONJ occurrence. In the fivefold cross-validated multivariate logistic regression, elastic net, RF, and SVM models, the mean AUROC value (0.78) across 100 random iterations revealed the favorable performance of these models.

VEGF is one of the most important growth factors for regulating vascular development and angiogenesis—it acts by stimulating proliferation of vascular endothelial cells and increasing vascular permeability [37]. The VEGF protein family includes VEGF-A (also known as VEGF), VEGF-B, VEGF-C, and VEGF-D, all of which modulate angiogenesis by binding to VEGF receptors such as VEGFR1, VEGFR2, and VEGFR3. Among interactions between VEGF and VEGFR, a well-known cellular response pathway is VEGF-A, which is the principal inducer of angiogenesis and VEGFR2 signaling [38]. During vascular formation, VEGF-A binds to VEGFR2 and activates multiple pathways through signaling intermediates. The stimulation of various downstream pathways by these interactions promotes endothelial proliferation and angiogenesis [38].

Angiogenesis affects the processes of bone repair and wound healing. It is known that suppression of osteoclasts by long-term bisphosphonate treatment can affect osteoblast function, thereby impairing bone repair. A study using mice with osteoblast-specific deletion of *VEGFA* showed that appropriate levels of VEGF were required for coupling angiogenesis and osteogenesis at repair sites [39]. Inhibition of VEGF signaling, which enhances intramembranous bone formation, has been shown to impair bone healing by interfering with the conversion of cartilage callus to bone callus [40]. It has also been reported that zoledronate therapy reduces VEGF levels and bone blood flow [41]. These results suggest that the anti-angiogenic activity of bisphosphonates could result in avascular necrosis and impair tissue repair.

The rs881858 SNP, a significant factor in our study, is located in a *VEGFA* intron. The function of this SNP has not been characterized, but chromosomal interactions between the rs881858 SNP region and the promoter of *VEGFA* may affect the regulation of *VEGFA* gene activity. Previous genomic studies have reported that patients with wild-type (G allele) rs881858 were associated with chronic kidney disease development due to decreased nephrogenesis, which is induced by reduced *VEGFA* activity [42,43]. In another publication, it is suggested that wild-type homozygotes have higher insulin resistance, indicating impaired angiogenesis [44].

Another SNP, rs10434, was found to have a marginally significant impact on BRONJ occurrence. The rs10434 polymorphism, which is located in the 3′UTR region of *VEGFA,* has been widely studied in association with carcinoma and pregnancy loss. A study of Chinese patients found that the rs10434 A allele was significantly associated with an increased risk of B cell chronic lymphocytic leukemia, ostensibly via the upregulation of a VEGF-based autocrine pathway [45]. Another study of Iranian pregnant women found that the recessive allele (G) of rs10434 was significantly more frequently encountered among patients with pre-eclampsia than among controls, possibly because of decreased VEGFA expression [46,47]. These results are consistent with our findings.

For environmental factors, numerous potential risk factors for BRONJ have been considered. Treatment duration, administration route, co-morbidities, co-medications, smoking, and age are among the most commonly reported potential risk factors for developing ONJ [48,49]. However, evidence is still sparse due to the lack of prospective studies. In this study, among demographic factors, age and treatment duration were significant risk factors for BRONJ. The significance of these demographic factors was expected, and our findings were consistent with those of previous studies [50,51].

In this study, various machine learning approaches were utilized to predict BRONJ. Regardless of the machine learning method used in this study, the AUROC values indicated that all of the models in this study performed well. In particular, the AUROC value from the multivariable logistic regression model was exactly the same as that from the elastic net, a penalized linear regression model that combines the penalties of the lasso and ridge methods [52]. Meanwhile, RF is an ensemble method of bootstrap aggregated binary classification trees. RF grows binary classification trees on the basis of bootstrapped samples of the training data while using only a random subset of available features at each node to find the optimal splitting rule [53,54,55]. Through repeating these processes, RF can generate thousands of decorrelated decision trees (i.e., the ensemble) that can provide more robust committee-type decisions. SVMs were implemented using linear and radial basis function kernels in this study. Linear kernel SVMs have a single tuning parameter, C, which is the cost parameter of the error term, whereas radial kernel SVMs have an additional hyperparameter that defines the variance of the Gaussian, i.e., how far a single training example’s radius of influence reaches [55,56].

This study had some limitations, including its small sample size, which led to an underpowered study. Due to the nature of osteoporosis, the number of men (*n* = 2) was so small that they were not included in this study to rule out the effect of gender. Some demographic factors such as smoking history and corticosteroid therapy could not deal with covariates because of insufficient information. It was possible to be additional potential confounders that were not eventually included in the predictive model. Additionally, we did not examine the underlying mechanism at the molecular level. Moreover, the lack of external validation and other factors that may affect the performance of machine learning algorithms also must be considered when interpreting the findings of this study. Nevertheless, the strength of this study is that this is the first study using machine learning methods to predict BRONJ. In addition, our control group consisted of well-defined patients by oral and maxillofacial surgeons after undergoing dentoalveolar surgery. In many other studies, it has been pointed out that inclusion of healthy subjects or uncertain controls in genetic studies results in bias.

## 5. Conclusions

To our knowledge, this was the first study to investigate the effects of variations in the *VEGFA* gene on BRONJ complications among patients with osteoporosis. Additionally, this study utilized machine learning approaches to predict BRONJ occurrence. Although further functional studies are needed to verify our findings, these results could contribute to clinical decision-making based on ONJ risk.

## Figures and Tables

**Table 1:** Patient characteristics of study patients.

Characteristics	Case (*n* = 58)	Control (*n* = 67)	*p*
Age (years)			0.003
<65	3 (5.2)	16 (24.2)	
≥65	55 (94.8)	50 (75.8)	
Comorbidity			
Hypertension	36 (62.1)	28 (41.8)	0.024
Diabetes mellitus	18 (31.0)	16 (23.9)	0.370
Cardiovascular disease	8 (13.8)	8 (11.9)	0.757
Rheumatoid arthritis	7 (12.1)	2 (3.0)	0.080
Thyroid disease	4 (6.9)	2 (3.0)	0.415
Kidney disease	2 (3.4)	3 (4.5)	1.000
Liver disease	0 (0)	2 (3.0)	0.499
Cancer	2 (3.5)	6 (9.1)	0.284
Treatment duration (months)			
<36	13 (25.5)	30 (55.6)	0.002
≥36	38 (74.5)	24 (44.4)	

**Table 2:** Associations of genotypes with bisphosphonate-related osteonecrosis of the jaw.

Gene Polymorphism	Allele Change	Minor Allele Frequency	Grouped Genotypes	Case (*n* = 58)	Control (*n* = 67)	*p*
rs699947	A > C	0.253	AA, AC	22 (37.9)	31 (46.3)	0.347
			CC	36 (62.1)	36 (53.7)	
rs2010963	C > G	0.439	CC	14 (25.0)	8 (12.5)	0.077
			CG, GG	42 (75.0)	56 (87.5)	
rs25648	C > T	0.081	CC	51 (87.9)	52 (77.6)	0.131
			CT, TT	7 (12.1)	15 (22.4)	
rs3024987	C > T	0.211	CC, CT	56 (96.6)	63 (94.0)	0.685
			TT	2 (3.4)	4 (6.0)	
rs3025022	C > T	0.181	CC, CT	18 (31.0)	23 (34.3)	0.696
			TT	40 (69.0)	44 (65.7)	
rs3025035	C > T	0.202	CC	34 (59.6)	49 (73.1)	0.246
			CT, TT	23 (40.4)	18 (26.9)	
rs3025039	C > T	0.133	CC	42 (72.4)	50 (74.6)	1.000
			CT, TT	16 (27.6)	17 (25.4)	
rs10434	A > G	0.113	AA, AG	7 (12.1)	18 (26.9)	0.039
			GG	51 (87.9)	49 (73.1)	
rs998584	C > A	0.421	CC	7 (12.1)	14 (21.2)	0.176
			CA, AA	51 (87.9)	52 (78.8)	
rs6905288	G > A	0.240	GG, GA	21 (36.2)	33 (49.3)	0.142
			AA	37 (63.8)	34 (50.7)	
rs881858	G > A	0.133	GG, GA	18 (31.0)	10 (14.9)	0.031
			AA	40 (69.0)	57 (85.1)	

**Table 3:** Multivariate analysis to identify predictors of bisphosphonate-related osteonecrosis of the jaw.

Variables	Crude Odds Ratio (95% CI)	Adjusted Odds Ratio (95% CI)	Attributable Risk (%)
Age ≥ 65 years	5.87 (1.61–21.34) **	16.05 (1.87–138.05) *	93.8
Treatment duration ≥ 36 months	3.65 (1.60–8.36) **	3.67 (1.36–9.94) *	72.8
*VEGFA*			
rs10434, GG	2.68 (1.03–6.97) *	3.16 (0.97–10.31)	68.4
rs881858, GG/GA	2.56 (1.07–6.14) *	6.45 (1.69–24.65) **	84.5

Logistic regression analysis with backward elimination was carried out with variables such as age, hypertension, treatment duration, rs10434, and rs881858. * *p* < 0.05, ** *p* < 0.01.

## Data Availability

The data presented in this study are available upon reasonable request from the corresponding author.

## Abbreviations

AUROC	Area under the receiver-operating curve
BRONJ	Bisphosphonate-related osteonecrosis
CI	Confidence interval
ONJ	Osteonecrosis of the jaw
OR	Odds ratio
RF	Random forest
SNP	Single nucleotide polymorphism
SVM	Support vector machine
VEGF-A	Vascular endothelial growth factor A
